# Registerial Adaptation vs. Innovation Across Situational Contexts: 18th Century Women in Transition

**DOI:** 10.3389/frai.2021.609970

**Published:** 2021-06-01

**Authors:** Stefania Degaetano-Ortlieb, Tanja Säily, Yuri Bizzoni

**Affiliations:** ^1^Department of Language Science and Technology, Saarland University, Saarbrücken, Germany; ^2^Department of Languages, Faculty of Arts, University of Helsinki, Helsinki, Finland

**Keywords:** linguistic innovation, register variation, gender-specific linguistic variation, diachronic variation in language use, periods of change in language use, computational sociolinguistics, Late Modern English, historical sociolinguistics

## Abstract

Endeavors to computationally model language variation and change are ever increasing. While analyses of recent diachronic trends are frequently conducted, long-term trends accounting for sociolinguistic variation are less well-studied. Our work sheds light on the temporal dynamics of language use of British 18th century women as a group in transition across two situational contexts. Our findings reveal that in formal contexts women adapt to register conventions, while in informal contexts they act as innovators of change in language use influencing others. While adopted from other disciplines, our methods inform (historical) sociolinguistic work in novel ways. These methods include diachronic periodization by Kullback-Leibler divergence to determine periods of change and relevant features of variation, and event cascades as influencer models.

## 1. Introduction

The investigation of the temporal dynamics of language is becoming a growing field outside of historical linguistics. Computational modeling (such as distributional, probabilistic, neural, etc.) is adopted to trace diachronic developments. From a computational sociolinguistic perspective, our aim is to apply computational models to shed light on how sociolinguistic factors are involved in the temporal dynamics of language use. We look at Late Modern British English in the 18th century, a period of transition in social terms, considering register and gender, with a special focus on changes in women's language use. Consider, for instance, O'Brien (2009)'s examination of female writers, centered upon an analysis of the ways in which women “deployed and refashioned” (p. 2) enlightenment concepts of gender, constructing a discourse that defined and defended female intellectual and moral agency, and in the longer term enabled the development of 19th-century feminist discourse (cf. Carr, 2009, review no. 831).

As one sociolinguistic factor, *register* is known to impact language use (Biber, 1988; Halliday, 1989; Biber et al., 1999) and has been accounted for in historical linguistic analyses (Nevalainen and Raumolin-Brunberg, 2003, p. 195). Registers are defined as clusters of associated lexico-grammatical features having a greater-than-random tendency to co-occur (Halliday, 1988, p. 162) and are referred to as language use according to the situational context. In sociolinguistic terms, social interaction is realized in linguistic forms through meanings, i.e., the social context is realized by specific lexico-grammatical choices. This relation is bidirectional, i.e., a particular social context influences the lexico-grammatical choices made, while at the same time lexico-grammatical choices create a social context. However, registers are not static; in fact, studies on register formation processes have shown how registers emerge and evolve over time due to changing social contexts (Ure, 1971, 1982). The process of modernization within a society, for example, is one major trigger leading to register change and the formation of new registers (Halliday, 1988; Degaetano-Ortlieb, 2015; Teich et al., 2016). Previous research into register variation in the history of English has found that speech-based and popular written registers have been going through a gradual process of colloquialization, where they have drifted toward more oral styles, while expository “specialist” registers have developed toward the literate end of the continuum (Biber and Finegan, 1997). This research, however, has ignored other social factors (e.g., gender, social class), which alongside the situational context have a major impact on language use (Argamon et al., 2003; Nevalainen and Raumolin-Brunberg, 2003; Säily, 2016, 2018a).

*Gender* is one of these social factors having an impact on change in language use. As is customary in sociolinguistic research, we use the term gender rather than sex to denote this variable sociocultural construct. Previous studies have shown how the language use of women and men is distinctively involved in change, women often leading the change of the investigated linguistic phenomena (Nevalainen and Raumolin-Brunberg, 2003). Our chief interest is in the language use of women of the middle and upper classes, a social group in transition in the 18th century. According to Ylivuori (2019, p. 39, 43), the notion of gender was in flux at that time, with the early modern idea of gender as a cline between men and women being replaced by the idea of two separate genders, which encouraged heterosociability as women were thought to be naturally polite and thus to act as an improving influence upon men. This was especially the case among the upper and middle classes, whose men and women began to spend more time together in public and whose ideal of marriage also changed toward a more affectionate and informal relationship (Hay and Rogers, 1997, p. 41, 18–24). Paradoxically, women's “natural” femininity was regarded as something that required education and constant repetition in order to stick, and what exactly constituted feminine was up for debate, which gave women of the “better sort” some leeway to negotiate how they spoke and behaved, as well as opportunities to gain a better education and claim some power (Ylivuori, 2019, p. 45ff.; cf. Tieken-Boon van Ostade, 2010). We analyze the language use of these women in two different situational contexts: court trials and letter writing.

Given register and gender, we formulate the following main hypotheses:

H1 *Registerial adaptation*: due to language-external pressures in more formal contexts (court), middle and upper-class women will linguistically adapt to more formal conventions diachronically to meet social pressure (cf. Degaetano-Ortlieb, 2018)
H2 *Registerial innovation*: in less formal contexts (letters to family members), women will indicate a different linguistic behavior, perhaps even leading the change toward a more oral or involved style (cf. Säily et al., 2017b)

In our approach, we take into account the following considerations. First, similar to other studies (Gries and Hilpert, 2010), we want to broaden our understanding of the temporal dynamics in language use by considering linguistic factors as well as more than one extra-linguistic factor (here: time, register, and gender). Second, for decades in historical linguistics two things have been mainly assumed: (1) linguistic domains/levels are relatively modular and discrete, and (2) time periods are relatively fixed (cf. Nevalainen and Traugott, 2012, p. 3). These assumptions are increasingly being challenged—most prominently by those exploring the probabilistic nature of language (Bod et al., 2003; Halliday, 2004), and also due to the application of statistical methods and data mining techniques to the analysis of temporal dynamics in language (Gries and Hilpert, 2010; Degaetano-Ortlieb and Teich, 2018, 2019).

Considering the first point raised by Nevalainen and Traugott (2012, p. 3), while previous work on diachronic variation has mainly focused on one linguistic level [e.g., phonology; see also sociolinguistic (Labov, 1994, 2001) and computational sociolinguistic studies (e.g., Eisenstein, 2015; Nguyen et al., 2016)], recent studies are increasingly considering several linguistic levels and possible interplay across linguistic levels in order to obtain a more comprehensive picture of change (Bermudez-Otero and Trousdale, 2012; Broccias, 2012; Degaetano-Ortlieb and Teich, 2019; Bizzoni et al., 2020).

As for the second point, analyzing and comparing fixed time periods by pre-defining historical stages has been the standard practice (e.g., Kytö, 1993; Nevalainen and Raumolin-Brunberg, 2003; Degaetano-Ortlieb, 2015, 2018; Teich et al., 2016; Säily et al., 2017a; Degaetano-Ortlieb et al., 2019c). The rise in interest in the investigation of temporal dynamics of cultural sociolinguistic phenomena has triggered a whole wave of more exploratory, data-driven approaches targeted toward determining when particular changes occur rather than comparing predefined periods. For example, Gries and Hilpert (2008) propose a specific clustering approach to analyze the development of English targeted at single linguistic phenomena, van Hulle and Kestemont (2016) use stylometric methods to periodize literary works of Beckett, and Popescu and Strapparava (2013) characterize epochs by a statistical approach. We have designed a data-driven periodization technique based on Kullback-Leibler divergence (henceforth KLD) that allows us to detect actual periods of change from the data itself, not confined to a particular linguistic phenomenon, but across linguistic levels (Degaetano-Ortlieb and Teich, 2018, 2019). Formally, KLD measures how much two probability distributions (here: one for future and one for past language use) diverge from one another. High KLD indicates high divergence, i.e., future and past language use diverges, while low KLD indicates periods of consolidation where future and past are relatively similar to each other. Thus, peaks in KLD point us to periods of change. Moreover, interest is rising within the computational sociolinguistic community in detecting influencer (initiators of changes) and influenced (those adopting changes) groups. Recently, event cascades have shown promising results on social media interactions (Dutta et al., 2020) and conversations (Daw et al., 2020). We adapt event cascades to model long-term diachrony.

Methodologically, we start by considering baseline models encompassing all language users of both registers (letters, court trials), comparing models of lexis, grammar, and morphology by KLD over time. We then proceed to compare gender-specific models over time. In line with H1, we assume converging trends for the more formal context (court trials), i.e., diachronically language is used more similarly across social groups, while we assume less converging trends for the informal context of family letters. To capture H2, we focus on the informal register using event cascades to investigate whether particular social groups influence others (e.g., women influencing men). Finally, we qualitatively inspect changes in the letter corpus in the broader context of the 18th century as a period of transition for women.

## 2. Related Work

### 2.1. Historical Sociolinguistics

Linguistic variation and change is often socially conditioned. Sociolinguistic research has discovered, for instance, that women tend to lead language change (Tagliamonte, 2012, p. 63). Present-day sociolinguistics has typically relied on apparent-time studies of change, which make the problematic assumption that people do not change their language use as they get older. Historical sociolinguistics, spearheaded by Nevalainen and Raumolin-Brunberg (1996, 2003), has enabled the study of language change in real time in the long diachrony. It has also moderated the finding of women leading changes by pointing out that historical facts like women's lack of access to certain registers have limited their involvement in some changes, which have been led from above by men (Nevalainen and Raumolin-Brunberg, 2003, p. 131). This seems likely to also apply to stylistic change in courtroom discourse, where female attendees would have formed a small minority (Emsley et al., 2018a). Considering research on innovation and propagation, Peter Petré's pioneering work (Petré and Van de Velde, 2018; Petré and Anthonissen, 2020) shows how individual variation and diachronic change are related. Petré et al's. work combines qualitative and quantitative methods to measure the degree of grammaticalization at the level of individual attestations of particular grammatical features, also tracing lifespan change in individual authors.

While most of the research within variationist/quantitative sociolinguistics, whether present-day or historical, has focused on individual linguistic features (e.g., Tagliamonte, 2009; Nevalainen et al., 2018), there are also some large-scale studies that take a bird's-eye view of sociolinguistic variation and change in a specific corpus. These studies have typically utilized either keyword analysis or, more frequently, part-of-speech ratios (Rayson et al., 1997; Markus, 2001; Heylighen and Dewaele, 2002; Argamon et al., 2003; Newman et al., 2008; Säily et al., 2011, 2017b; Bamman et al., 2014). This work has revealed interesting and surprisingly consistent patterns of gender variation over time: whereas men's style is often characterized by an informational focus and a high frequency of e.g., nouns, determiners and numerals, women's style tends to be more oral and exhibits greater writer and addressee involvement, as evidenced by the high frequency of such features as first- and second-person pronouns, verbs, negations, and interjections (cf. Biber and Burges, 2000; Vartiainen et al., 2013). An increasing frequency of involvement features can also be seen in the colloquialization of some genres, such as personal letters, which in the fifteenth to the seventeenth centuries seems to have been led by the upper social ranks, as they “could increasingly afford to write simply to keep in touch with friends and family, for which a more oral, involved style would be in order” (Säily et al., 2017b, p. 38). The new POS-tagged version of the *Corpus of Early English Correspondence Extension*, which is equipped with social metadata, enables such research to be conducted in eighteenth-century data as well (see further 3.1.2 below).

In their studies of a number of linguistic changes in eighteenth-century English correspondence, Nevalainen et al. (2018) found that many but not all of the changes were led by women and that most of the consistently conservative individuals were men, thus supporting the sociolinguistic finding of female advantage in language change (Nevalainen, 2018, p. 257–259; Säily, 2018b, p. 242). Some of the changes they analyzed were connected to an involved style of writing, such as the incoming progressive aspect and the increase in the “embodied attribute or trait” meaning of the nominal suffixes -*ness* and -*ity*, as in *your kindness*. The latter was argued by Säily (2018a, p. 214–215) to support the claim made by McIntosh (1998, 2008, p. 231) that British culture in the later eighteenth century underwent a process of “feminization,” by which McIntosh referred to an increasing concern with the feminine values of politeness and sensibility amongst those aspiring to belong to the upper echelons of society (see also Ylivuori, 2019). This could imply that middle- and upper-class men emulated the language use of the increasingly well-educated women of the same classes, who authored publications and hosted literary salons (Myers, 1990; Pohl and Schellenberg, 2003; Tieken-Boon van Ostade, 2010)—at least in some registers (cf. McIntosh, 1998). On the other hand, these women also became able to catch up with men's more nominal style and adapt it to their own purposes, which could have been reflected in the correspondence of the eighteenth-century literati (McIntosh, 1998, p. 205; Säily, 2018a). While so far considered in isolation, this study combines both views, moving toward a more comprehensive picture of the temporal dynamics involved.

### 2.2. Register Studies

Registers are referred to as language use according to the situational context and are defined by clusters of associated linguistic features having a greater-than-random tendency to co-occur (Halliday, 1988, p. 162). Similarly, Ferguson (1994, p. 16) states that a register is characterized by “the linguistic differences that correlate with different occasions of use.” While there have been many definitions of registers with similar notions and register studies have a long tradition in linguistics (Biber, 1988; Halliday, 1988; Martin, 1992; Ferguson, 1994), “the computational study of linguistic registers was a niche area and received little attention in computational work on language overall” (Argamon, 2019). Computational work on register studies has relied on the probabilistic notion of feature co-occurrences, e.g., using classification to categorize texts according to register based on register-indicative features (e.g., Atkinson, 1992; Biber and Conrad, 2001; Argamon et al., 2008; Eisenstein et al., 2011; Teich et al., 2013, 2016), to automatically annotate register labels to account for register differences in text analysis tasks (Giesbrecht and Evert, 2009; Sharoff et al., 2010), to improve information retrieval (Morato et al., 2003; Freund et al., 2006), and for register-sensitive text generation (e.g., Reiter and Williams, 2010; Crystal, 2011; Ficler and Goldberg, 2017; Jhamtani et al., 2017).

Driven by a more theoretical perspective, there have been studies on the acquisition of registerial knowledge (Ravid and Tolchinsky, 2002; Ravid and Berman, 2009). Language users acquire knowledge on using language appropriately in particular situations by mapping relevant linguistic forms to the context of communication, considering the range of expressive options available to them. “Mastery of register appropriateness thus plays an important role in acquisition of communicative competence [...]” and well-educated individuals command a wide range of registers (Ravid and Berman, 2009, p. 2). Moreover, there have been studies on register formation and evolution using corpus-based to more exploratory data mining techniques (e.g., Ferrara et al., 1991; Biber and Finegan, 1997; Nowson et al., 2005; Herring and Paolillo, 2006; Argamon et al., 2008; Teich and Fankhauser, 2010; Teich et al., 2016; Degaetano-Ortlieb and Teich, 2019; Degaetano-Ortlieb et al., 2019a). As these studies show, register is one among many factors influencing language use, such as time, medium (written vs. spoken), and other sociolinguistic variables (e.g., age, gender), which symbiotically affect each other.

In our own previous work considering time, gender and register, we have separately shown that in formal contexts (court proceedings) middle- and upper-class women linguistically adapt to male language use at the lexical and grammatical levels (Degaetano-Ortlieb, 2018), while in less formal contexts women maintain, and possibly lead changes to, a more oral or involved style (Säily et al., 2011, 2017b; Säily, 2018a) considering the grammatical and morphological levels.

Following up on this line of research, we use exploratory data-driven methods to investigate across the linguistic levels of lexis, grammar, and morphology, our two hypotheses of gender-specific *registerial adaptation* in a formal register and gender-specific *registerial innovation* in an informal register, comparing language use of women to men across court proceedings and letters to family members.

### 2.3. Computational Modeling

#### 2.3.1. Detecting Periods of Change

Borrowed from mathematics and applied in engineering fields, Kullback-Leibler divergence's popularity is growing across humanities fields as diverse as stylistics, literary studies, history, and linguistics as a measure for modeling variation. For example, Hughes et al. (2012) measure stylistic influence in the evolution of literature, Klingenstein et al. (2014) analyze language use in criminal trials, Bochkarev et al. (2014) use KLD comparing word distributions within and across languages, Pechenick et al. (2015) analyze cultural and linguistic evolution, and Fankhauser et al. (2014) demonstrate the applicability of KLD for corpus comparison at large. In our own work, we have used KLD to analyze the linguistic development of English scientific writing over time^1^ (Degaetano-Ortlieb and Teich, 2016; Degaetano-Ortlieb and Strötgen, 2018; Degaetano-Ortlieb et al., 2019b), to investigate intra-textual variation across sections of research papers from genetics (Degaetano-Ortlieb and Teich, 2017), to analyze scientifization effects in literary studies (Degaetano-Ortlieb and Piper, 2019), to detect typical features of history texts (Degaetano-Ortlieb et al., 2019c), and to investigate gender- and class-specific changes in court proceedings of the Old Bailey Court (Degaetano-Ortlieb, 2018).

With our novel method of data-driven periodization, we address a common challenge in diachronic analysis: to *determine* periods of change rather than using pre-defined periods. This is an endeavor pursued in various disciplines, such as biology, musicology, literary studies, and marketing research, as well as socio- and historical linguistics, among others. In the latter, as Nevalainen and Traugott (2012, p. 3) point out, rather than using pre-defined fixed periods to analyze linguistic diachronic change, one seeks to detect *when* changes occur on a continuous scale. In musicology, for example, to detect periods of stylistic change in popular music, Mauch et al. (2015) apply data-driven methods from bioinformatics on pre-selected audio features. In literary studies, van Hulle and Kestemont (2016) use stylometric methods with selected function words for periodization of particular prose texts. Gries and Hilpert (2008) use Variability-based Neighbor Clustering algorithms to determine periods of change for selected linguistic features [*get*-passives, verb conjugation suffixes *-(e)th* and *-(e)s*]. Ji (2010) applies Hierarchical Cluster Analysis to a corpus of Chinese focusing on selected morpho-syntactic patterns underpinning the evolution of Chinese lexis. Recently, Belinkov et al. (2019) applied periodization based on word embeddings on the Arabic portion of the OpenITI corpus^2^ following Gries and Hilpert (2008)'s VNC algorithm and adapting it to Word-Embedding-based Neighbor Clustering (WENC). Most similar to our work is Barron et al. (2018)'s study of parliamentary debates on the French Revolution applying overall KLD to sequential speeches and considering how much speeches diverge over time. In our work on the linguistic development of English scientific writing, in addition to considering overall KLD tendencies, we inspect features contributing to periods of increased divergence (cf. Degaetano-Ortlieb and Teich, 2018, 2019) enabling us to analyze reasons for and effects of change.

#### 2.3.2. Modeling Influencer Groups

Considering our second hypothesis, H2, of registerial innovation in less formal contexts, we are also interested in detecting which sociolinguistic groups might initiate a change and whether it is adopted by other groups. For this, we use the Multivariate Hawkes Process (Hawkes, 1971; Allan, 1976), often employed to model time-bound series, such as share trends (Hawkes, 2018) and earthquake shocks (Yuan et al., 2019), as *event cascades*, and recently also used in sociolinguistics to model turn-taking interactions in social media (Goel et al., 2016; Zhang et al., 2018; Dutta et al., 2020), as well as conversations (Daw et al., 2020). We adapt event cascades to model longer diachronic influencing trends.

## 3. Materials and Methods

### 3.1. Data

#### 3.1.1. The Old Bailey Corpus (OBC)

To depict the formal register of court proceedings, we use the *Old Bailey Corpus* (OBC; Huber et al., 2016) based on proceedings of the Old Bailey Court in London. These proceedings contain transcribed utterances of the court's trials spanning from 1674 to 1913. According to Emsley et al. (2018b) the City of London “required that the publisher should provide a ‘true, fair and perfect narrative’ of the trials” and in particular they state that “witness testimony is the most fully reported element of the trials.” Thus, the witness utterances can arguably be seen as a relatively precise account of spoken English of that period (cf. Huber, 2007 on the precision of the corpus as a whole). Therefore, we opt to consider the victims' and witnesses' utterances only, excluding lawyers, judges, interpreters, and defendants. The OBC was built from a digitized version of the proceedings representing a balanced subset.

In terms of annotation, utterances and sociolinguistic information was identified semi-automatically. This procedure was quite time-consuming and definitely not a trivial task. In fact, Huber and his team developed a dedicated annotation tool which allowed them, first, to automatically detect speakers based on a list of 7,500 male and female first names (about 95% coverage), and second, to scroll through the data searching for sociobiographical information to be annotated. Witness utterances, for example, started with statements about the profession of the speakers (cf. Huber et al., 2016).

Extra-linguistic information contains speaker information including gender, age, occupation (according to the HISCO standard), social class (HISCLASS standard), speaker role (defendant, interpreter, judge, lawyer, victim, and witness), and textual information (scribe, printer, publisher). Linguistic annotation is provided at the token, lemma, and part-of-speech levels using the CLAWS7 tagset (reported accuracy of 94–95%). The version used in our studies amounts to about 14 million words and is encoded in CQP (Evert, 2005). We focus on the middle-/upper-class subcorpus according to the HISCLASS standard, with 171,084 tokens for women and 1,370,390 for men. The OBC is available through a CQPweb platform.^3^

#### 3.1.2. Tagged Corpus of Early English Correspondence Extension (TCEECE)

The *Corpora of Early English Correspondence* (CEEC; Nevalainen et al., 1998–2006) were compiled to facilitate research in historical sociolinguistics. The genre of personal letters was chosen by the compilers for two reasons. Firstly, letters are a “speech-like” genre (Culpeper and Kytö, 2010, p. 17) resembling spoken conversation, which is the primary medium of social interaction and hence of interest to sociolinguists, who see it as the hotbed of change. Secondly, correspondence is a genre that would have been available to anyone who was literate, which means that by focusing on letters it was possible to achieve a wider social representativeness than by selecting texts written for publication, which would mostly have been authored by highly educated men. Nevertheless, a bias toward these men is evident even in the CEEC: they were the most literate social group, and their letters were considered important enough to be preserved and later edited. The corpora are based on published original-spelling editions of letters, which were sampled and digitized by the corpus team. This approach enabled the collection of millions of words of text but has the drawback of copyright issues, due to which only part of the corpora have been published. The extra-linguistic information on the social background of the informants, compiled by the team based on the editions as well as other historical and biographical sources, includes, e.g., gender, social rank, domicile, and the relationship between the writer and recipient of the letter (Raumolin-Brunberg and Nevalainen, 2007). About a quarter of the informants are women.

While the original corpus covered the period of c.1410–1681, its eighteenth-century *Extension* (CEECE) extends the end date until 1800. In the present study, we use the POS-tagged version of the CEECE, or TCEECE, which comprises about 2.2 million words in 4,923 letters sent by more than 300 individual writers. Prior to POS-tagging it with CLAWS, the spelling of the corpus was normalized using VARD (Baron, 2011a,b) along with some additional manual normalization, including abbreviations; however, as the normalization only targeted sufficiently frequent items, some orthographic variation still remains. The accuracy of the POS-tagging with the CLAWS5 tagset is c. 94.7% (Saario and Säily, 2020). We use a subcorpus of the TCEECE consisting of letters written between nuclear family members, which provides an interesting counterpoint to the formal speech-based register represented by the OBC. To match the OBC data, we further narrow down the corpus by focusing on men and women of the middling and upper social ranks during the time period 1720–1799. The size of this subcorpus is about 500,000 words, of which women's letters comprise 38.8% (cf. Kaislaniemi, 2018, p. 56).

### 3.2. Modeling Variation

#### 3.2.1. Measuring Divergence Between Language Uses

Recently, research in linguistic variation and change has increasingly relied on information-theoretic approaches. In particular, relative entropy formalized as Kullback-Leibler divergence (Kullback and Leibler, 1951) has proven effective to measure divergence between two probability distributions, *A* and *B*, derived from linguistic feature sets (cf. Fankhauser et al., 2014 using words). In our case, we consider three types of linguistic feature sets: lexical (word), syntactic (part-of-speech tag), and morphological (suffix). Given these levels, we define a feature set viewing a corpus as being realized as a probability distribution at one of these linguistic levels. Basically, KLD measures the number of additional bits needed to encode a given distribution *A* with another distribution *B* given a set of features (see Equation 1). Note that the set of features used with KLD can be quite vast.


(1) $$\begin{array}{l} D\left(A\|B\right)=\underset{i}{\sum }p\left(feature_{i}|A\right)log_{2}\frac{p\left(feature_{i}|A\right)}{p\left(feature_{i}|B\right)} \end{array}$$

The probability of the *i*th linguistic feature (e.g., a word or suffix) in *A*, *p*(*feature*_*i*_|*A*), and the *i*th feature's probability in *B*, *p*(*feature*_*i*_|*B*), are used to measure the amount of additional bits needed. The sum over all features gives an overall divergence measure, namely KLD *D*(*A*||*B*), which is always positive. The higher the KLD for *A* given *B*, the more the two distributions diverge. In addition, Jelinek-Mercer smoothing is used (lambda at 0.05; cf. Zhai and Lafferty, 2004; Fankhauser et al., 2014) to assign a non-zero probability to unseen features and improve the accuracy of feature probability estimation. In Degaetano-Ortlieb and Teich (2019, section 3.2.1), we give a detailed explanatory description of KLD based on a concrete calculation example.

Moreover, KLD is an asymmetric measure. Thus, the directionality of a comparison matters, i.e., *A* given *B* might result in a different value than *B* given *A*. This is particularly useful when considering language use. For example, a layperson might well be understood by an expert (e.g., in patient-doctor conversations), *D*(*patient*||*doctor*), while an expert's language use might be difficult for a layperson to understand, if the expert uses his/her usual field-specific language (e.g., a doctor using specialized medical terminology), *D*(*doctor*||*patient*).

Besides an overall indication of divergence between *A* and *B*, we can also inspect the individual feature weights, calculated by pointwise KLD (see Equation 2).^4^ This allows us to inspect which features are primarily associated with a divergence, i.e., those features needing a (relatively) high amount of additional bits for encoding, and thus, strongly contributing to variation between *A* and *B*.


(2) $$\begin{array}{l} D_{f}\left(A\|B\right)=p\left(feature_{i}|A\right)log_{2}\frac{p\left(feature_{i}|A\right)}{p\left(feature_{i}|B\right)} \end{array}$$

The feature weights obtained from KLD can be directly interpreted as bits of information. The more bits a feature needs to be encoded, the more typical it is for *A* in comparison to *B* and can thus be determined to be a relevant feature of variation for *A* when compared to *B*.

#### 3.2.2. Data-Driven Periodization

Based on KLD, we have developed a novel data-driven periodization to determine periods of change. The approach has the following components: (1) comparison of adjacent years by KLD for the linguistic levels selected, (2) relatively unconstrained feature selection across linguistic levels, and (3) inspection of features involved in change with high contribution to the overall divergence (KLD).

For feature selection, we opt to have a relatively unconstrained selection, i.e., rather than preselecting linguistic features known to be possibly involved in change, we use ngram sequences. In particular, we consider the lexical level by selecting all words (unigrams), the grammatical level by selecting pos-trigram sequences, and the morphological level based on a list of suffixes. The choice for trigrams was made after experimenting with different ngram sizes: bigrams proved to be too short to depict phrase/clause structure, and fourgrams and fivegrams are too long, leading to sparse data. In fact, pos-trigrams have also proved to work well in other diachronic studies (Culpeper and Kytö, 2010; Kopaczyk, 2013; Degaetano-Ortlieb and Teich, 2016; Degaetano-Ortlieb et al., 2019a). For suffix selection and extraction, we rely on experts' linguistic knowledge. In total, we consider 30 suffixes^5^ selected by manually revising extracted lists from the corpora to ensure data quality. Note that any kind of linguistic unit could be used for comparison (e.g., phoneme, morpheme, word, etc.).

**Figure 1:**
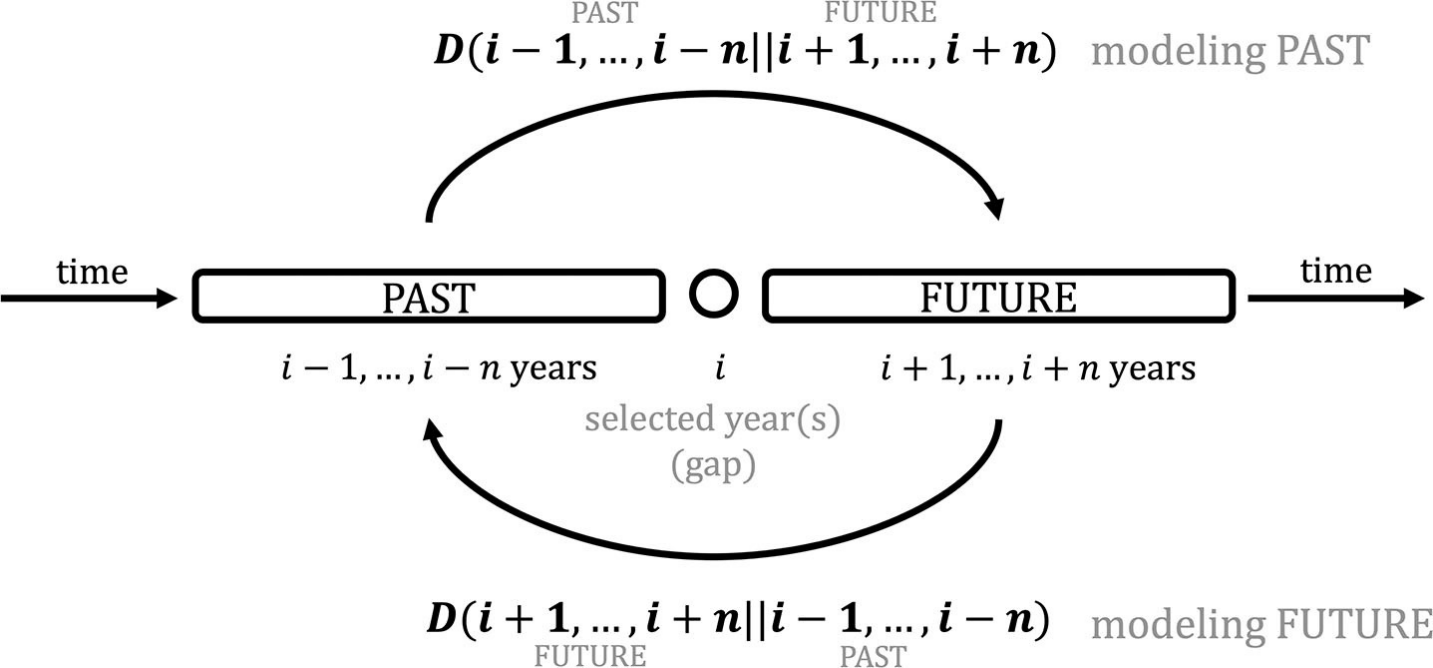
Data-driven periodization with Kullback-Leibler Divergence.

Comparison of adjacent years by KLD is illustrated in Figure 1. Basically, we slide over the timeline, comparing a range of years preceding and following a selected year with KLD. This allows us to find peaks and troughs in KLD which indicate a change. The procedure is operationalized as follows (also illustrated in Figure 1):

(1) Select a year *i* (or range of years, if the publication is not yearly) as a gap and a window size *n* of preceding (PAST: *i* − 1, ..., *i* − *n*) and following (FUTURE: *i* + 1, ..., *i* + *n*) years (e.g., 20 years);
(2) Calculate KLD for the PAST and FUTURE in both directions, i.e., divergence for a language model of the PAST given a language model of the FUTURE, *D*(*PAST*||*FUTURE*), and divergence for FUTURE given PAST, *D*(*FUTURE*||*PAST*);
(3) Slide to the next year and repeat (2).

In Degaetano-Ortlieb and Teich (2018), we experiment with different window sizes, showing how more fine-grained selections help to detect more subtle changes (e.g., a window size of 5 years), while coarser selections (e.g., a window size of 20 years, chosen here as it is assumed to cover a generation) lead one to inspect more general trends. Considering directionality, modeling PAST given FUTURE allows us to inspect outdated language use, and modeling FUTURE given PAST, more innovative language use.

Iterating over the years, we obtain a curve of KLD values showing a trend line for past language use as well as future language use. Peaks in KLD indicate periods of change; troughs point to periods of consolidation where the past and future are more similar to each other. This allows us to inspect at which particular point in time changes occur.

In addition to these overall trends, investigating individual feature contribution allows us to gain more profound insights into the kinds of change in the indicated periods. As we are dealing here with multiple bi-class comparisons, i.e., for one direction (e.g., FUTURE given PAST) at each gap one comparison of 20-year windows across ~40–60 years, one has to carefully choose how to inspect the many feature rankings in a meaningful way. One option is to inspect which features show high variation in their contribution, e.g., words having a high contribution to KLD only at particular points in time. For this, a standard deviation calculated across the feature rankings can be used. Another way of inspecting relevant features across comparisons is by ranking based on the feature weights of one particular year, allowing us to inspect more confined year-specific trends.

#### 3.2.3. Detecting Influencer Groups by Event Cascades

**Figure 2:**
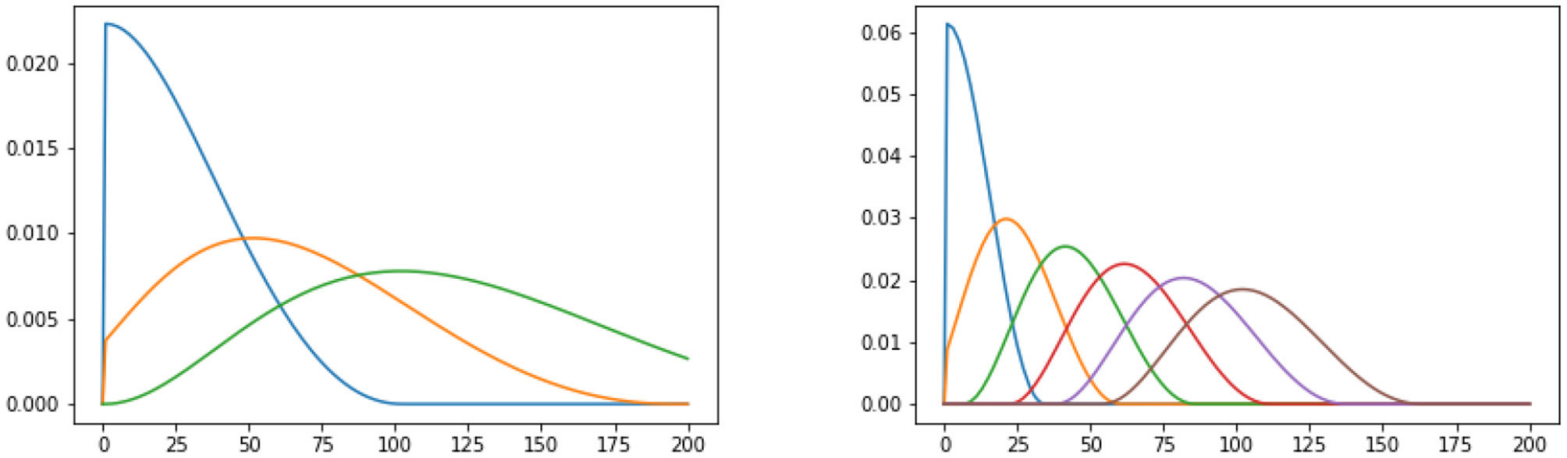
An abstract self-exciting cascade with 3 (left) and 6 (right) bases, or impulses. Impulse duration on the x-axis and intensity on the y-axis. Each impulse influences the intensity and duration of the next one. More impulses also mean faster and more intense impulses: the process is self-exciting.

Event cascades are a series of events marked on a temporal axis and having some form of self-exciting pattern (see Figure 2). In simpler terms, they are sequences of events happening with some form of domino effect. For example, the first shock of an earthquake happens randomly, while the succession of after-shocks happens only as a consequence of the first one, i.e., the first shock initiates a cascade of events. In conversations, one influential actor can start a change (a topic, a grammatical pattern), which others take up and re-use. The early and later adoptions of a new term on social media are a typical example of such kinds of cascades (cf. Eisenstein, 2019).

Since we are essentially trying to understand who influences whom in a social network, the goal of our model is to estimate the parameters α_*i*→*j*_ for all *i*,*j* in the population (Linderman and Adams, 2014). The excitation parameters can thus be represented as a matrix with α_*i*→*j*_, showing the excitation intensity of events from source *i* to target *j* (see Equation 3). In turn, each node in the network is taken as a source and target of the others:


(3) $$\begin{array}{l} \lambda_{t}^{\left(j\right)}=\lambda_{0}^{\left(j\right)}+\underset{e:t_{e}^{\prime }<t}{\sum }\alpha_{s_{e}\to j}\kappa_{s_{e}\to j}\left(t-t_{e}^{\prime }\right), \end{array}$$

where $\lambda_{t}^{\left(j\right)}$ is the excitement intensity function on the node *j* from all other nodes at time *t* (how much other nodes have influence on *j* at time *t*), α is a scalar excitement parameter, *s*_*e*_ indicates the source of event *e*, and κ_*s*_*e*_→*j*_ is a kernel decay function monotonically decreasing through time, constrained to integrate to 1 over positive arguments—the further away in time, the weaker the influence is expected to be.

The fundamental idea is that in a multi-party dialog, some speakers have a higher degree of influence on the style of the others: they start “event cascades” in the conversation. If actor *X* in a conversation repeatedly starts a topic or particular linguistic use, subsequently used by *Y* and *Z*, we may assume an influence from *X* to *Y* and *Z*—although this is not necessarily the case.^6^ The final result is a matrix of influencers and influenced. In our case, we model female and male language use of different time periods as different actors, i.e., the data is modeled as a series of concatenated pair-wise interactions between women and men sequentially within the same period. We divide periods into 20 years to match our KLD analysis. For example, women in the 1740–1759 period are modeled with men of the same period.

**Figure 3:**
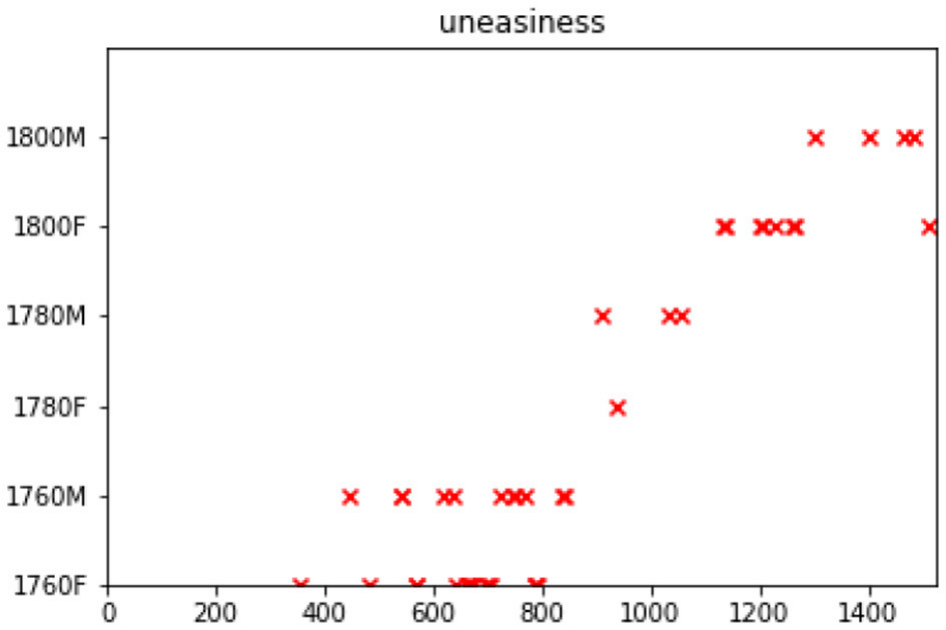
An event cascade for the word *uneasiness*. Sociolinguistic groups on the y-axis, chronologically ordered series of letters on the x-axis.

The event cascade's goal is to measure the intensity of the influence of *i* on *j* for a specific time interval Δ*t* and is modeled as a sum over *B* simple basis models (Equation 4), as the ones in Figure 2:


(4) $$\begin{array}{l} \kappa_{i\to j}\left(\Delta t\right)=\overset{B}{\underset{b=1}{\sum }}g_{b}^{\left(i\to j\right)}\phi_{b}\left(\Delta t\right). \end{array}$$

where ϕ_*b*_(Δ*t*) is the basis model (an impulse function that sums to one) and *g*_*b*_ are the dyad-specific weights over the basis models.^7^ This allows us to see whether one sociolinguistic group in the exchange has a particular influence over the others. The cascades these exchanges produce look like the ones in Figure 3, showing how words (such as *uneasiness*) are introduced in earlier periods and then continue to be used later on. Here, we can observe women starting the trend (1760F), influencing also both men and women of later periods. To obtain an overall impression of influencer and influenced groups, we use a heatmap visualization based on Equation (4) which shows the intensity of influence across groups.

## 4. Investigating Gender-Specific Registerial Adaptation and Innovation

### 4.1. Diachronic Tendencies Across Registers and Linguistic Levels

Using diachronic periodization with KLD, we start comparing diachronic baseline models of both the court and letter corpora (focusing on the middle and upper classes) across lexis, grammar, and morphology.

**Figure 4:**
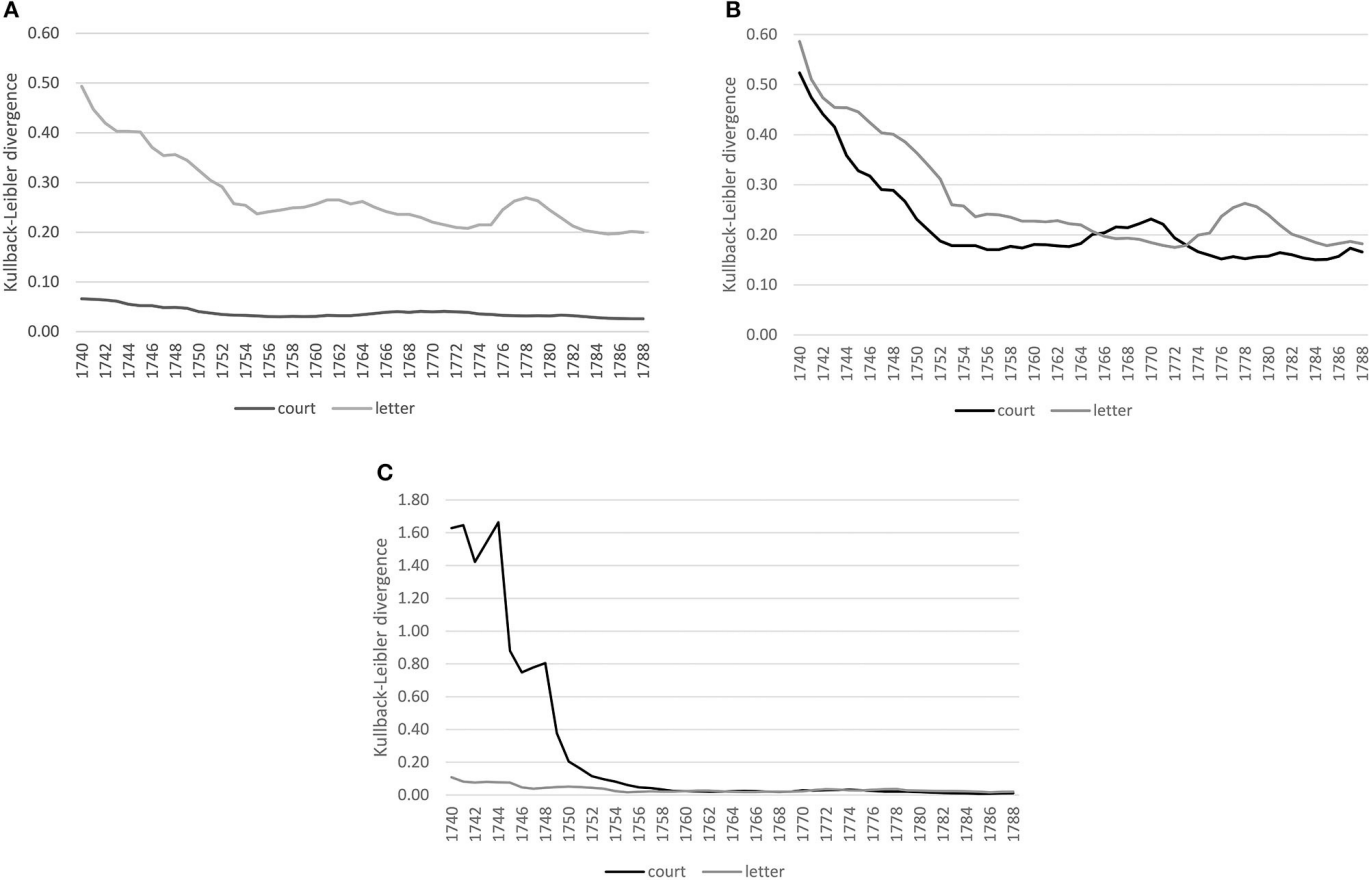
Baseline models for the court and letter corpora across linguistic levels. **(A)** Lexis (word-unigram). **(B)** Grammar (part-of-speech trigrams). **(C)** Morphology (suffixes).

Figure 4 shows converging trends over time for both corpora. For lexis (Figure 4A), the order of magnitude of the decrease varies across registers, the court proceedings showing lower divergences compared to the letters, indicating a more consolidated vocabulary in court in comparison to a more varied vocabulary in the letters, which, however, might also be an effect of spelling variation still present in the letter corpus for less frequent lexemes.^8^ At the grammatical level (Figure 4B), both registers show a similar decreasing trend, with convergence around the mid-1750s. At the morphological level (Figure 4C), the strong decrease in the 1740s for the court corpus is related to data sparsity in the preceding years used for modeling, but basically for both corpora the trend is a converging one.

The converging trend across linguistic levels is in line with previous work on the evolution of genres, registers and styles in English. For example, Claridge (2012, p. 82, 90) has shown trends toward standardization and regularization during 18th century English, arguing that enforcing and maintaining standardization is one of the functions of written, published language and that these written usages could then also promote the standardization of the spoken language (Milroy and Milroy, 1991, p. 35, 60, 64). Here, we see similar trends for a formal spoken register (court proceedings) and an informal written register (letters to family members). Considering a communicative perspective, there is evidence that these converging tendencies across registers are related to the fact that language users strive for efficient communication. Convergence is one effect leading to achieve this goal. Consider results from Degaetano-Ortlieb and Teich (2019) and Bizzoni et al. (2020), who show a converging tendency in scientific writing for 17th–18th century English at both the lexical and the grammatical level. They argue that a decrease in variation, i.e., convergence on particular options, is beneficial for communication. The entropy, i.e., the uncertainty about which linguistic items to use (in terms of production) or expect (in comprehension), is reduced and shared conventionalized options arise over time among language users. Despite change in language use related to converging trends leading to conventionalization, change is clearly also brought about by innovations. However, De Smet (2016) shows how conventionalization is a precondition of innovation. One aspect that is still understudied is how innovations come about and who leads those changes.

Taking up a sociolinguistic perspective considering gender, we further investigate how gender-specific groups might change their language use over time across registers, something not captured by the baseline models as they comprise all language users. We focus on middle- and upper-class women and men.

### 4.2. Gender-Specific Diachronic Tendencies

Here, we investigate our two hypotheses of gender-specific *registerial adaptation* in the formal contexts of court proceedings (H1), and *registerial innovation* in the informal setting of letters to family members (H2), both across lexis, grammar, and morphology. Diachronic periodization is used to determine periods of change and derive relevant features of variation. Event cascade models are applied to determine gender-specific influencer groups. Detailed micro-analytical sociolinguistic inspections are presented to elaborate on the computational findings.

#### 4.2.1. Lexis

**Figure 5:**
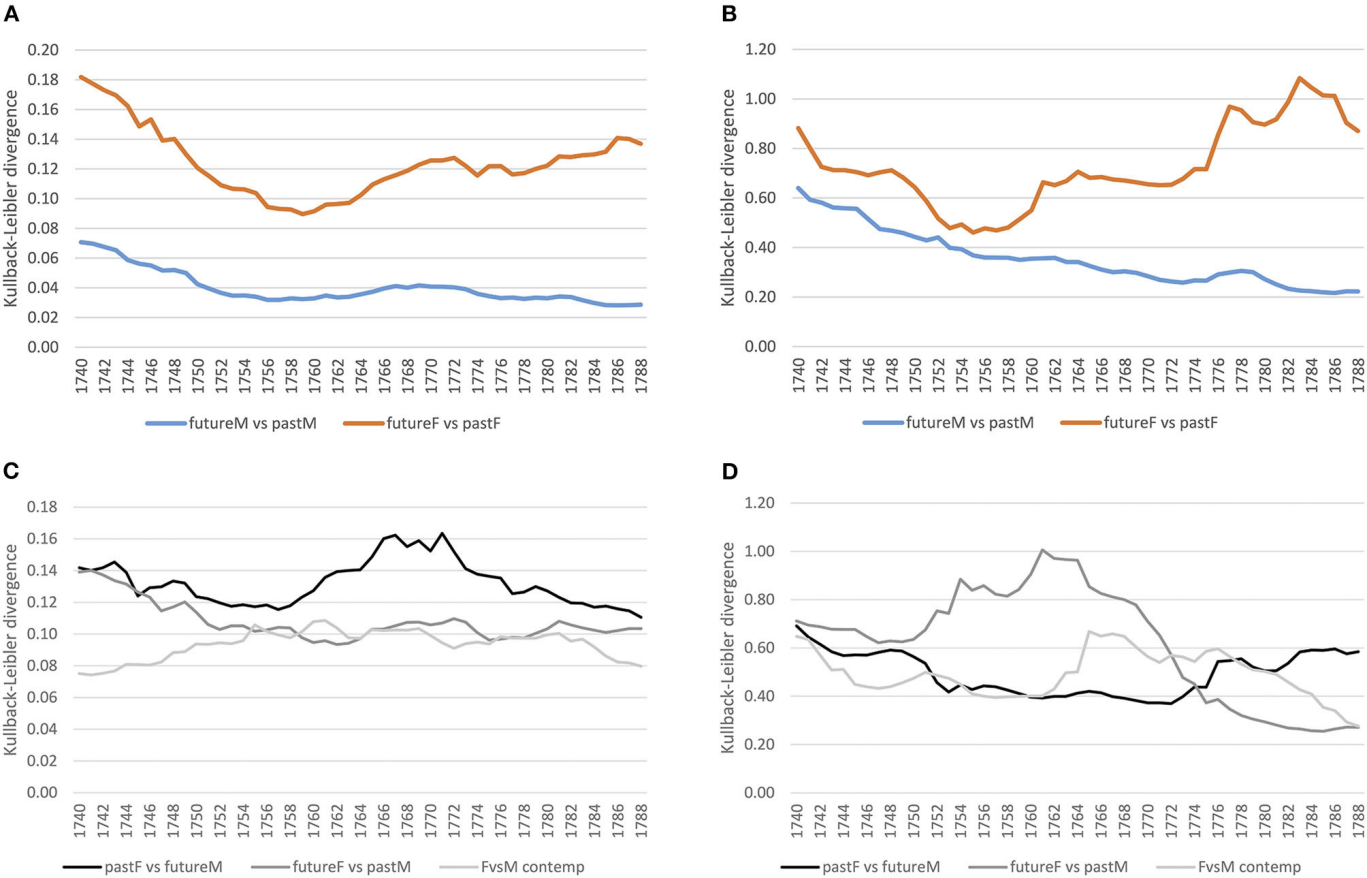
Gender distinguished, diachronic and contemporary models (court vs. letter) at the lexical level (word-unigram). **(A)** Court: within gender comparison. **(B)** Letter: within gender comparison. **(C)** Court: diachronic and contemporary comparison. **(D)** Letter: diachronic and contemporary comparison.

First, we inspect how language use of the future has changed from past language use for both the court and letter corpora at the lexical level. For this, we select 1 year, and compare the preceding 20 with the following 20 years using KLD. This is done for all years, sliding over the timeline toward the future (see section 3.2.2). Figures 5A,B show how male language use converges over time in both registers. Female language use seems to converge at first, until the mid-1750s/60s, with an increasing diverging tendency afterwards. Thus, women change their language use over time, while male language use increasingly converges—a tendency that applies to both registers.

Second, we ask how different the language use of women vs. men is for the same period of time (contemporary models). For this, we compare across gender the same 20 years by KLD, sliding again over the timeline toward the future. Figure 5C shows that in the court setting, contemporary female and male language use (see the light gray line) diverges increasingly until the mid-1750s and stabilizes afterwards with small ups and downs. Comparing this to the letter corpus (see Figure 5D, light gray line), the contemporary model converges at first until the end of the 1750s, diverges in the 1760s/70s, and converges further in the 1780s. In both registers, this seems to reflect ongoing change at the lexical level between male and female language use.

Third, we can inspect how female language use diverges from or converges to male language use of the future and past. This is an important perspective, because change is ongoing, so a contemporary model will miss possible adaptation tendencies. For example, if future female language use converges with past male language use, then women might have adapted to language use of males in court after having been possibly exposed to that language for a while. If this is the case, and as we have seen that future and past male language use converges over time (see Figure 5A), at some point past women and future men should converge as well. While social pressure of conforming to particular conventions might arise in the court setting, in the informal setting of letters to family members we assume a less stable converging trend.

Comparing both registers, we clearly see registerial differences. First, considering past female vs. future male language use in the formal court setting (see black line in Figure 5C), there is a rise in divergence around the 1760s,^9^ while in the informal letter setting (see Figure 5D), divergence is relatively stable. Second, considering future female vs. past male language use (dark gray line), in the court corpus divergence decreases continuously, stabilizing around the 1750s, while in the letter corpus there is a peak in divergence from the 1750s to the mid-1760s with a steep decrease afterwards. In conclusion, in court we see registerial adaptation as women change their language use converging to men,^10^ while in the letter corpus change points to registerial innovation, future women diverging from the past. Note that innovation takes place within a particular time period (the peak in Figure 5D), after which women and men converge again. Men, instead, seem to be more conservative (relatively stable divergence of pastF vs. futureM).

**Figure 6:**
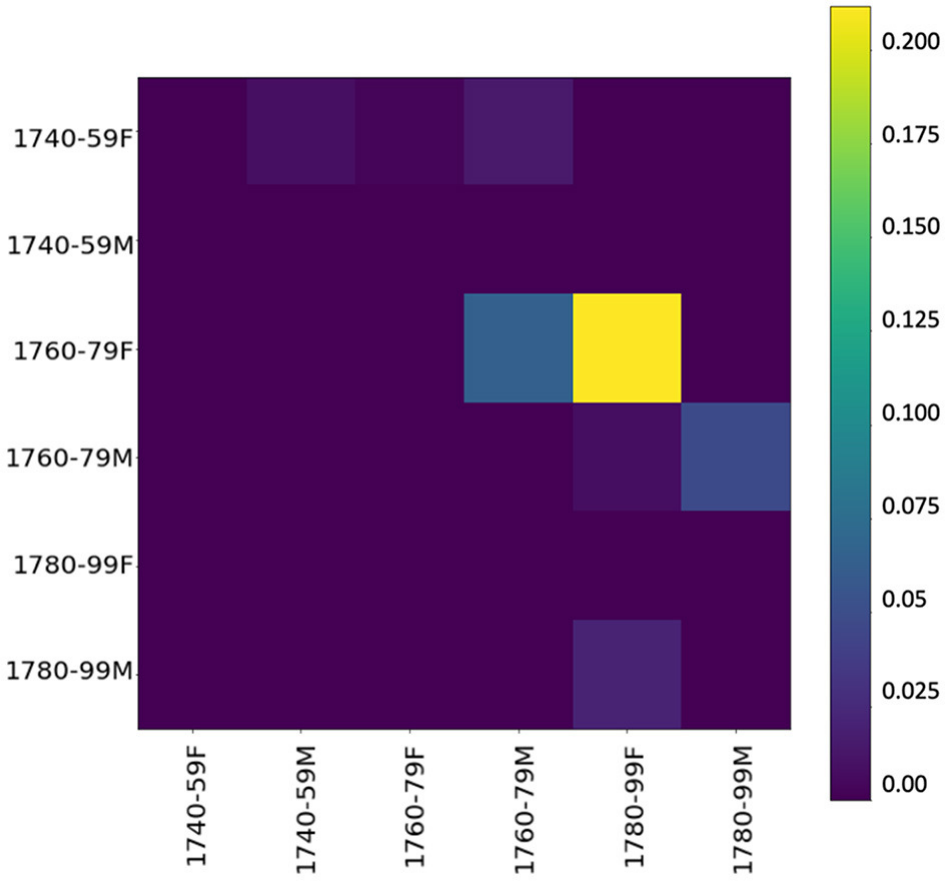
Influencer heatmap of event cascades over time at the lexical level (500 most frequent words).

To inspect whether, during the innovative period, one gender's language use has an influence on the other's, we use event cascades (see section 3.2.3),^11^ considering far-reaching influences (over the whole dataset), which allow us to see that influences tend to cascade down to the next period. Figure 6 shows the influencing gender-and-period groups on the y-axis and the influenced groups on the x-axis—the higher the value, the stronger the influence. Basically, if a group (e.g., females 1740–1759) starts several cascades, it has a relevant influence. The yellow square in the heatmap shows a strong chronological influence by women of the 1760/70s on women of the following period. A less strong but still visible influence is on men of the same period as well as the following period, confirming our assumption derived from KLD tendencies in Figure 5D. Especially in the central period (ranging from 1760 to 1779), women appear to have a tendency to start using new words, both in the functional and in the content realm. Thus, it is mainly female language use at the lexical level (including both content and function words) in the informal setting of letters to family members that influences male language use.

##### 4.2.1.1. Micro-Analysis: Lexis

**Figure 7:**
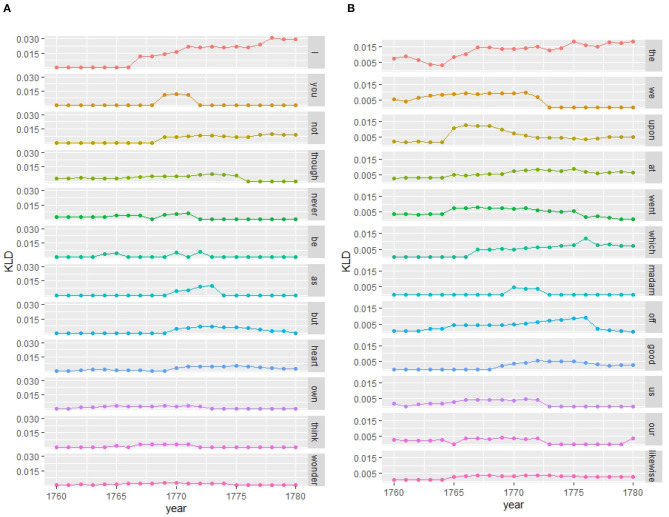
Features contributing to period of change (from top to bottom showing highest to lowest contribution)—contemporary model women vs. men (word-unigram). **(A)** Women. **(B)** Men.

As we have seen distinct language use of women and men in the period of the 1760s and 1770s (see peak in the contemporary model in Figure 5D, and heatmap of event cascade in Figure 6), we look at the features that contribute to an increase in KLD in the contemporary model. Comparing Figures 7A,B, we clearly see a more involved personal style for women than for men. Women distinctively use in comparison to men the personal pronouns *I* and *you*, negation (*not, never*) and conjunctions of contrast and concession (*though, but*) as well as mental verbs (*think, wonder*); see example (1). These features also indicate a more verbal style of writing. Men, instead, distinctively use the determiner *the*, prepositions (*upon, at*), and the relativizer *which* pointing to nominal style as well as first-person plural pronouns (*we, our, us*); (2). Whereas (1), written by a wife to her husband, clearly concentrates on interpersonal relationships using affective language and ego- and addressee-involvement, example (2) from a husband's letter focuses on narrating what he has done or observed with a third party, using exclusive *we*, which cannot in this context be regarded as an involvement feature. Thus, middle- and upper-class family letters in this period exhibit the oft-observed distinction between personally involved women and informatively oriented men, while the register as a whole moves in the involved direction led by women (cf. the “feminization” of McIntosh, 1998), shown also in the cascade analysis.

(1) **I** declare **I** should be rejoiced, was there no occasion, to write on things of more consequence, **as I never** wish to give **you** vexation, however my Duty to your Son obliges me to speak sometimes of things **I** know **you** don't like to hear and yet in fact your **own** interest is concern'd **as** much **as** his, **I** mean in regard to the payment of your Sisters Fortune—**I never think** of it **but** it leaves a dead weight on my **Heart**, and **I** cant help saying that it is a most cruel thing in **you** to keep runing up the interest **as you** do […]
  (Eliza Taylor née Pierce to her husband, Thomas, January 29, 1766; PIERCE_028)^12^
(2) Palmer & I took **our** horses on Friday & rode to **the** Town of Dock, 2 miles, & to **the** passage **which** I have marked. From thence **we** saild to Lord Edgcombes gates & walkd over a fine lawn to **the** house, **which** is about halfway up **the** hill. **The** stone of this country is too hard & rough to work to a truth, as **we** masons say. Its colour too, **which** is a reddish black. being all really marble mix'd with a very white lime, is not agreeable to **the** eye, & **the** house being old, with 4 octagons newly added to **the** angles, makes a better appearance **at** a distance than near.
  (Roger Newdigate to his wife, Sophia, October 17, 1762; NEWDIGA_037)

The female subcorpus in the 1760s–70s includes both lesser-known professionals and gentry like Mrs. Taylor in (1) and literary figures like Lady Mary Wortley Montagu, and their language use is in part surprisingly similar. McIntosh (1998, p. 205) argues that at the same time as more and more women became published authors, the social roles of women within the family became more restricted as the idealized “sentimental family” locked women inside the private sphere. We can see that Mrs. Taylor writes quite deferentially and considerately to her husband, while also committing the face-threatening act of trying to tell him what to do in a financial matter, over which she as a woman has no legal control. This is one of the contexts that seem to intensify the use of involvement features in family letters of the time, with women bringing up important issues while presenting themselves as loving wife-mothers driven by their feelings (cf. Ylivuori, 2019, p. 77–82). The feminized ideal of the sentimental family also influenced male writing, and even Sir Roger of example (2) has some affective and interpersonally involved passages in his letters to “My Dearest Sophy.”

#### 4.2.2. Grammar

**Figure 8:**
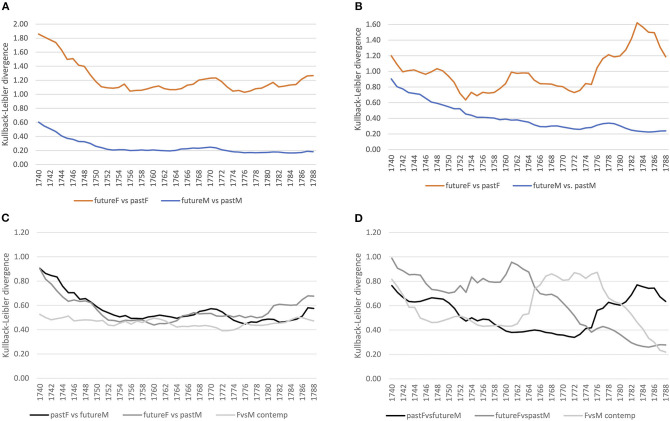
Gender distinguished, diachronic and contemporary models (court vs. letter) at the grammatical level (pos-trigrams). **(A)** Court: within gender comparison. **(B)** Letter: within gender comparison. **(C)** Court: diachronic and contemporary comparison. **(D)** Letter: diachronic and contemporary comparison.

Taking up the same approach for grammar as for the lexical level, we first compare female and male language use separately, considering future compared to past language use. In the formal court setting, we clearly see a converging tendency for both women and men, stabilizing around the 1750s (see Figure 8A). For the letter corpus, however, while male language use converges over time, women seem to converge at first until the mid-1750s, but increasingly diverge in their use of grammar afterwards compared to previous years (see Figure 8B). Thus, while men converge in the use of grammatical patterns in both settings, women change their use of grammar around the 1770s in the informal setting.

Considering contemporary language use of women and men in the court corpus, it is quite stable (see Figure 8C). The diachronic models of pastF vs. futureM and futureF vs. pastM confirm a relatively stable use of grammar in the formal setting. In the letter corpus, on the other hand, there is a period of change in the use of grammatical patterns around the mid-1760s/70s, where the language use of women and men differs as depicted by the peak in divergence. This period of change is also shown in the diachronic models: female language use before that period diverges from that of past men and past female language use diverges from that of future men after the period of change.

**Figure 9:**
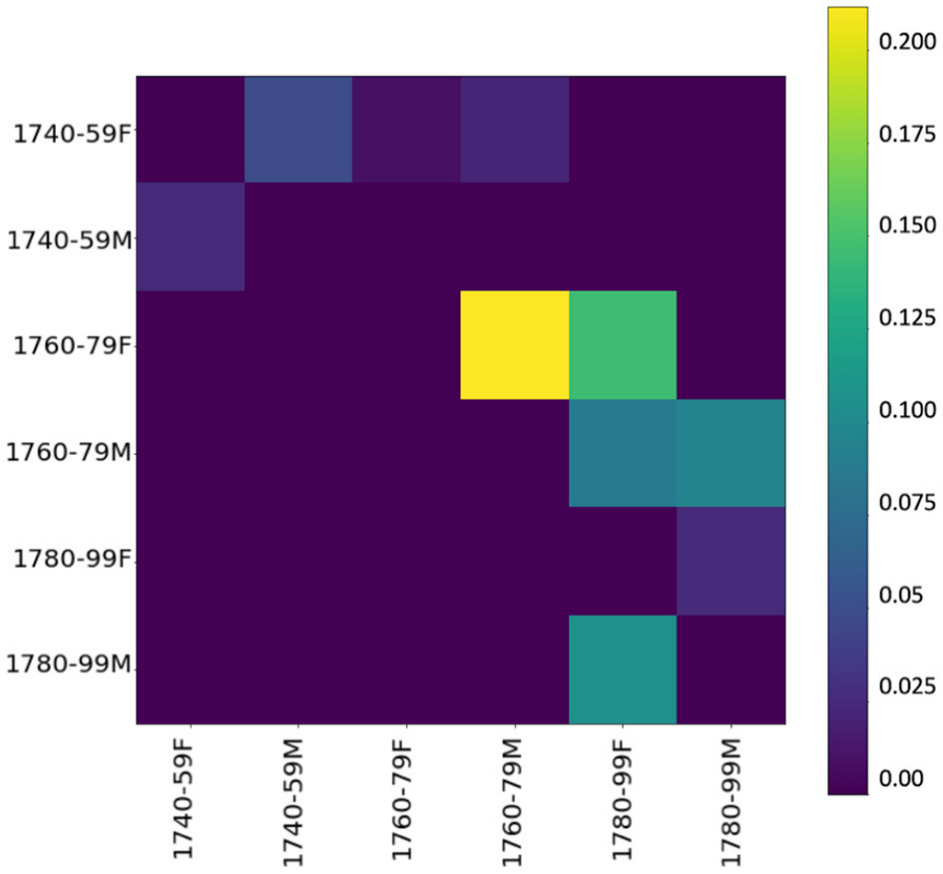
Influencer heatmap of event cascades over time at the grammatical level (pos-trigrams).

Looking at the event cascades for pos-trigrams (see Figure 9), again we can confirm an influencing trend of women toward men, especially in the period between 1760 and 1779. These results essentially mirror the findings at the lexical level, showing that the influence appears to go in the same direction, and with similar intensity, across both linguistic levels. Also, a grammatical influence of men over women is present, especially in the late period. This seems to indicate what has been shown in previous work: stylistic written features are adopted from men by women (cf. McIntosh, 1998, 205).

##### 4.2.2.1. Micro-Analysis: Grammar

**Figure 10:**
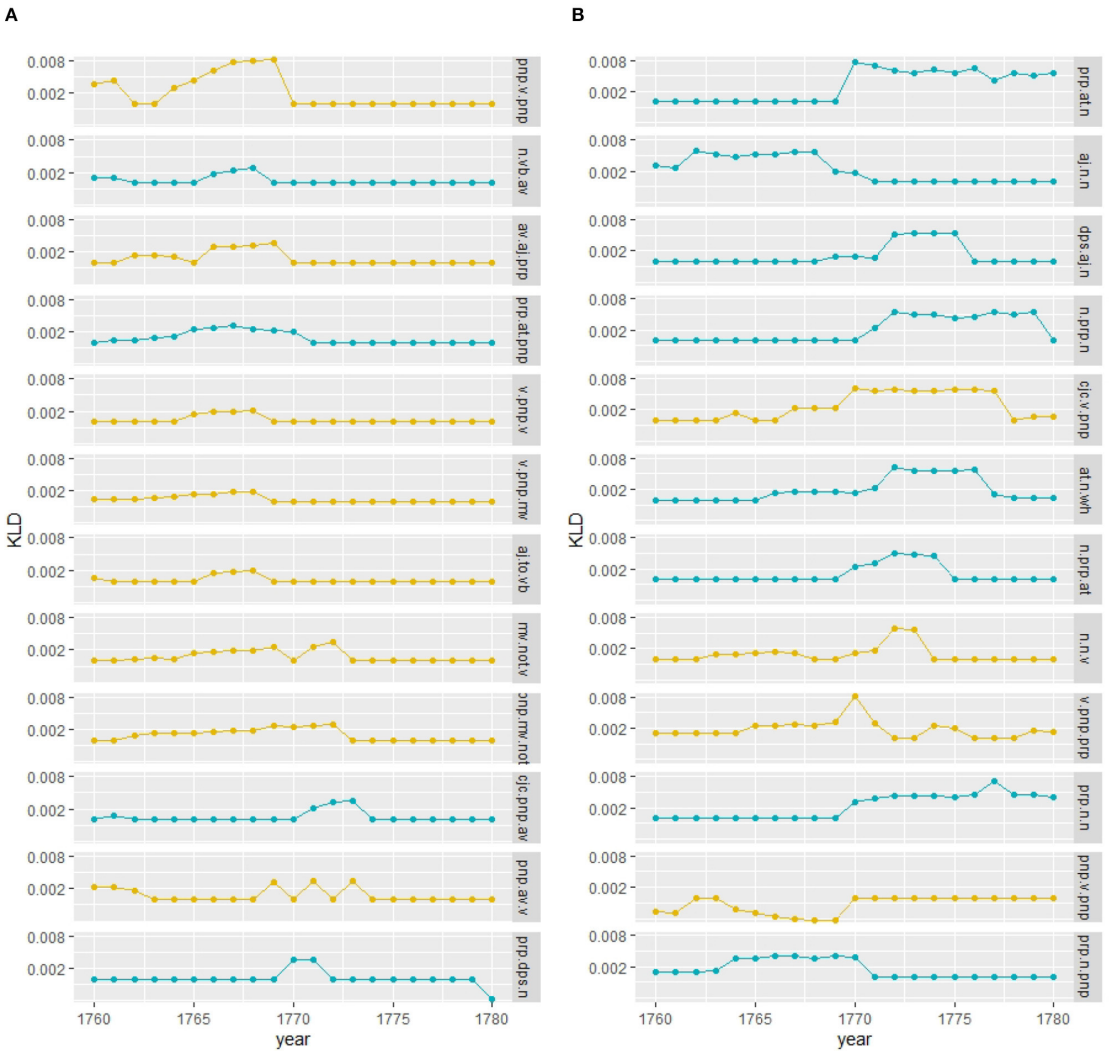
Features contributing to period of change (from top to bottom showing highest to lowest contribution; blue: nominal trigrams, yellow: verbal trigrams)—contemporary model women vs. men (pos-trigrams). **(A)** Women. **(B)** Men. aj, adjective; av, adverb; cjc, conjunction; at, determiner; mv, modal verb; n, noun; dps, possessive *s*/pronoun; prp, preposition; pnp, pronoun; v, verb; vb, verb *be*; wh, wh-word.

Inspecting features contributing to the period of change in the 1760s/70s for the contemporary model at the grammatical level (see Figure 8D), we see from Figure 10 that women distinctively use verbal style (yellow lines), while men rely on nominal style (blue lines). Matching the results at the lexical level, women make use of a personal involved style marked by grammatical patterns of first person pronouns combined with mental verbs [pronoun.verb.pronoun (pnp.v.pnp), such as *I wish/think/hope you*] as well as modality and negation [pronoun.modalverb.not (pnp.mv.not) such as *I can not bear, I should not have, I could not write*]; see example (3). The few nominal patterns also reflect the involved style of writing with evaluative patterns that often include intensifiers [adverb.adjective.preposition (av.aj.prp) such as *very useful/rude/kind to, very strange/painful for*; noun.be.adverb (n.vb.av) such as *topic is ever (interesting), stomach is so (weak)*; preposition.possessive.noun (prp.dps.n) such as *in my opinion/mind*]; (4).

(3) for I have such a fixed depression upon my spirits, that **I cannot** raise them to any decent degree of Chearfulness, - when I have told you the Cause, **I think** ***you***, at least, will not wonder at the Effect.
  (Frances Burney to her sister, Susanna, post - December 10, 1778; BURNEYF_011)
(4) Whoever is well acquainted with Venice must own it is the center of Pleasure, not so noisy, and **in my opinion** more refin'd than Paris. […] He is singular both in his manner and Sentiments, yet I am apt to beleive if he meets with a sensible Wife, she may be **very happy with** him.
  (Lady Mary Wortley Montagu to her daughter, Mary, Lady Bute, c. February 24, 1760; MONTAGU_192)

Men, instead, are distinguished by a very nominal and rather conventionalized style of writing using prepositional and compound patterns [e.g., preposition.determiner.noun (prp.at.n) as *to the queen, in the morning*, adjective.noun.noun (aj.n.n) as *small market town, tolerable drinking order*] used for narration of events, objects and places as well as verbal patterns of reporting [e.g., conjunction.verb.pronoun (cjc.v.pnp), such as *and said he, and told us*] and narration [e.g., verb.pronoun.preposition (v.pnp.prp) as *put him into, wrote it on, took us to* with material verbs; adverb.pronoun.verb (av.pnp.v) as *then they went, yesterday I sent, there we found* indicating place and time]. These patterns are illustrated in examples (5) and (6), written by two sons to their fathers; see also (2), where a husband narrates his activities using the adverb.pronoun.verb (av.pnp.v) pattern (*thence we saild*). Men (and boys; Pierce Taylor was a teenager at Eton) were often away from home and wrote about what they had seen and done, as well as general news of events in the places they were visiting. Even when women were traveling, like Lady Mary Wortley Montagu in (4), they tended to make their letters more about personal opinions and evaluation, or at least they used more explicit evaluative markers—compare (2), where Sir Roger Newdigate states his opinion about Lord Edgcombe's house but expresses it as a simple fact without hedging. These findings, then, match those at the lexical level, i.e., both linguistic levels reflect involved verbal language use of women strongly marked by modality, negation and evaluation vs. a more conventionalized nominal style of men in the informal setting focusing on narration of events, places and time.

(5) They **carried me to** the best houses **in the place**, shewed me whatever was worth seeing, and made several parties for me **in the country**.
  (Edward Gibbon to his father, Edward Sr, May 31, 1763; GIBBON_013)
(6) **When we came into the Play Fields** the Sixth Form went to the Doctor **and said we** would all return if he would make us a Promise of Oblivion, He said No, Mr Roberts took Grenville and lock'd him up, on which we gathered round his House.
  (Pierce Joseph Taylor to his father, Thomas, November 6, 1768; PIERCE_033)

#### 4.2.3. Morphology

**Figure 11:**
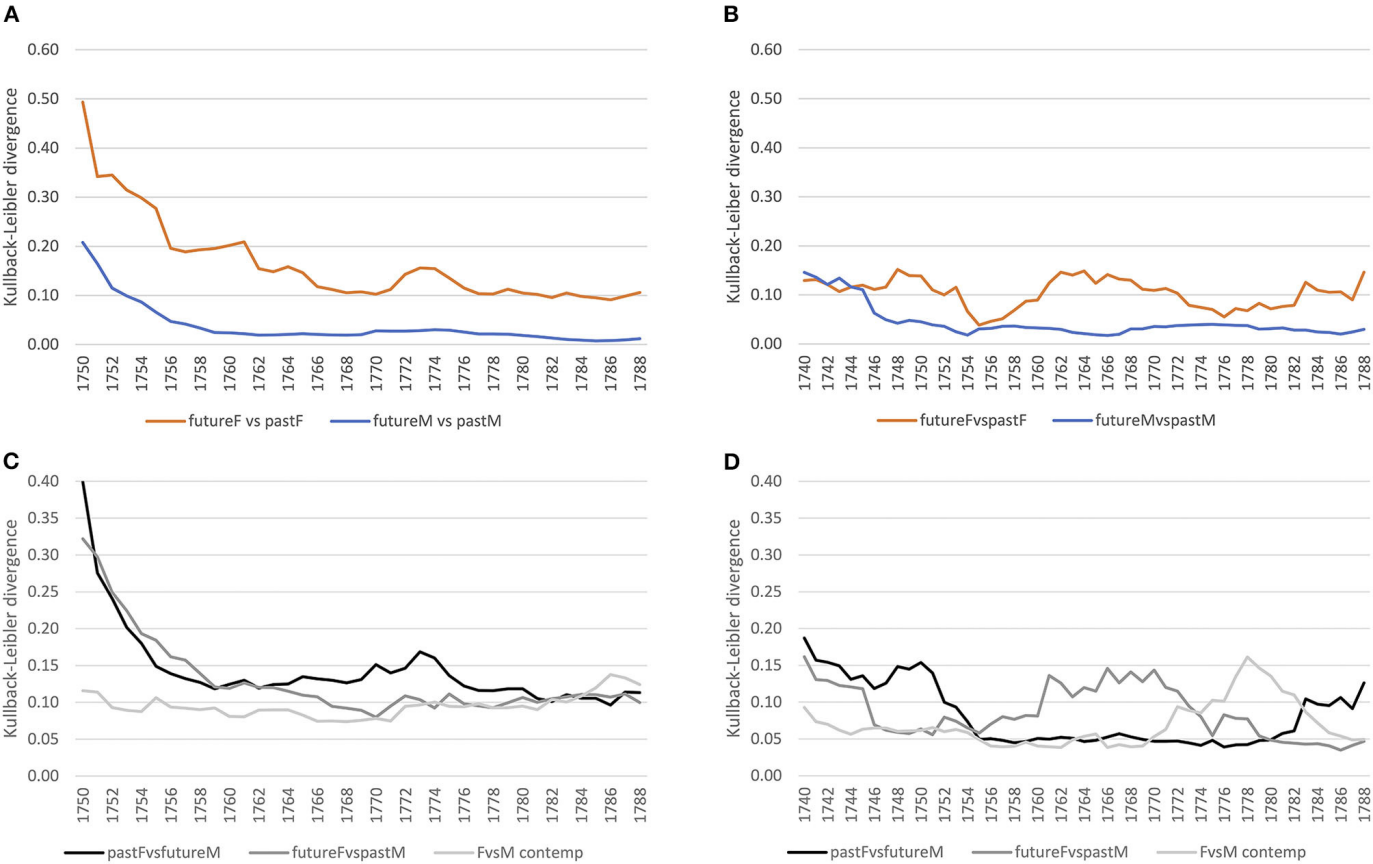
Gender distinguished, diachronic, and contemporary models (court vs. letter) at the morphological level (suffixes). **(A)** Court: within gender comparison. **(B)** Letter: within gender comparison. **(C)** Court: diachronic and contemporary comparison. **(D)** Letter: diachronic and contemporary comparison.

For the morphological level, we see similar converging trends within the same gender as for the lexical and grammatical levels for the court corpus (see Figure 11A). In the letter corpus, on the other hand, male language use converges, while female language use fluctuates compared to the past, indicating periods of ongoing change (see Figure 11B).

Considering the contemporary models, female and male language use is relatively stable in the court corpus (see Figure 11C), while in the letter corpus, there is a peak around the 1780s. This period of change is also depicted in the diachronic models: future women diverge around the 1760s from past men and past women diverge from future men by the mid-1780s.

**Figure 12:**
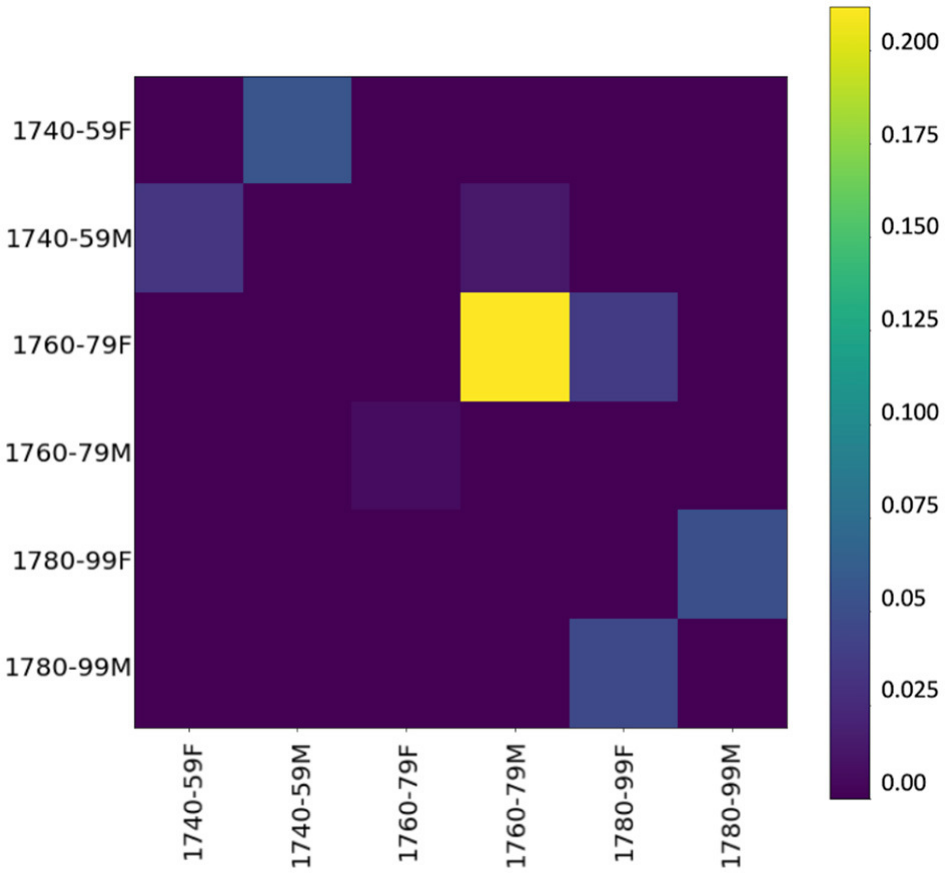
Influencer heatmap of event cascades over time at the morphological level (suffixes).

Our cascade model of influence (see Figure 12) confirms again that the influence of women over men is stronger than the other way around, especially in the period between the 1760s and the 1780s. Thus, starting from 1760 women introduce, rather than adopt, morphological innovations. Comparison with KLD results in Figure 11D shows that future female language use increasingly diverges from male language use of the past (dark gray line) in the 1750s. Thus, women seem to initiate a change adopted by men as shown in the cascade model.

In the last period, 1780–1799, divergence decreases in the contemporary model (see again Figure 11D). Here, unlike the other linguistic levels, the cascade model shows an influence of women and men on each other which also pertains to the last period.

##### 4.2.3.1. Micro-Analysis: Morphology

**Figure 13:**
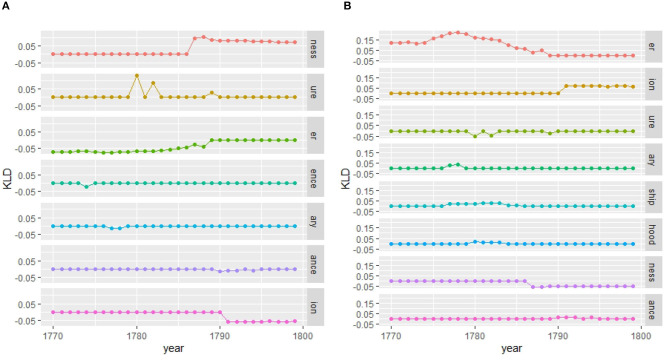
Features contributing to period of change (from top to bottom showing highest to lowest contribution)—contemporary model women vs. men (suffixes). **(A)** Women. **(B)** Men.

A micro-analytic inspection reveals that the peak in KLD around the 1780s is largely due to the nominal suffix -*ness* (see Figure 13). While no gender difference has been found in its productivity in eighteenth-century correspondence as a whole (Säily, 2018a), in the final 20-year period of the corpus we do find women using it highly productively in family letters. The most productive users were published authors: Frances Burney, Mary Wollstonecraft, and Hester Lynch Piozzi. As shown by Säily (2018a, 214), *- ness* in the eighteenth century was increasingly being used in the sense “embodied attribute or trait,” as in *your kindness*, and this change was led by women, potentially as part of the “feminization” of eighteenth-century culture (McIntosh, 2008, p. 231). Interestingly, while both men and women use the suffix in both positive and negative contexts, the types produced by women toward the end of the century are more skewed toward negative affect, as in (7).

(7) I hope God Almighty will preserve her to make us great Amends by her future Wisdom and Virtue for the Pain She now gives both to you and me by her **Grossness**, and her Contemptible Preference of the *Bon Ton and genteel Life* as She calls it, to every thing in this World and the next [...]

(Hester Lynch Piozzi to her daughter, Hester Maria Thrale, November 7, 1796; PIOZZI_061)

Another contributor to the peak around the 1780s is the nominal suffix -*ure*, again used most extensively by the same three women. As most of the types are borrowings from French, Latin or Italian with no base in English, this is not a case of increased productivity; however, the use of words with the foreign suffix may have been a way of signaling learnedness or sophistication, which would have been important to women like the intellectual Wollstonecraft or the Bluestockings Piozzi and Burney (Myers, 1990; Pohl and Schellenberg, 2003; Tieken-Boon van Ostade, 2010). Moreover, Piozzi married an Italian music teacher in the 1780s, while Burney later married a Frenchman, which may have influenced their use of -*ure* words, as in (8).

(8) M. d'Arblay has had a charming Letter from Comte Lally upon the ***brochure***—I intend also to enclose that, & dear Mr. Twining's, for your perusal, by Susanna.
  (Frances Burney to her father, Charles Burney, c. January 27, 1794; BURNEYF_027)

#### 4.2.4. Summarizing Temporal Dynamics of Gender-Specific Registerial Adaptation and Innovation

Overall, there is a converging trend in the formal setting of court proceedings across linguistic levels, pointing to registerial adaptation to formal conventions over time. Considering gender, the converging trend is steeper for women. In the informal setting of letters to family members, instead, across linguistic levels around the 1760s/70s/80s, women diverge from past language use of both women and men. Moreover, women lead changes which propagate to language use of later periods by men in particular. This clearly points to registerial innovation in the informal letter writing setting.

## 5. Summary and Discussion

In this work, we take a long-term diachronic perspective, investigating gender-specific differences in language use. Our focus is on women of the middle and upper classes as a social group in transition in this period, investigating change in language use across two different registers—one formal (court proceedings) and one informal (letters to family members). Thus, we consider two sociolinguistic factors (gender and register) involved in shaping the temporal dynamics of Late Modern British English in the 18th century. Computational methods have been used to model language use over time across three linguistic levels: lexis, grammar, and morphology.

We investigated two hypotheses: (a) *registerial adaptation* by middle- and upper-class women to formal conventions in the court setting, and, on the other hand, (b) *registerial innovation* in the informal setting of letters to family members. Findings have shown that underrepresented groups, such as women in the 18th century, can and do adapt to functional variation, such as registers, possibly triggered by external societal pressure. However, when no such pressure is at play, they create and shape new ways of using language and even lead changes which are then adopted not only by other women but also by men, who at that time enjoy a better social status.

These results could perhaps be seen to align with Labov's gender paradox, which states that “Women conform more closely than men to sociolinguistic norms that are overtly prescribed, but conform less than men when they are not” (Labov, 2001, p. 293). However, we cannot really state that women would conform *more* closely than men in the court setting, since we have established that they converge to men's language use, indicating that they are followers rather than leaders in the process of conventionalization there. The first part of the paradox would have been difficult to realize in the eighteenth-century courtroom since women had, by virtue of their gender, less access to the norms of this setting (see section 2 above). Therefore, we would not say that our results weaken Labov's claim *per se* but that its realization may depend on specific sociohistorical circumstances. Our results more unequivocally support the second part of the paradox as women innovate in the less tightly regulated setting of correspondence, which was also discovered by Nevalainen (2018, p. 258–259). In the eighteenth century, “letter-writing conventions became less formal, with their subject-matter including private as well as public matters, and letters were becoming an artistic, moral and intellectual literary form” (Somervell, 2011). Around the 1750s, the Bluestocking Society arose as a women's informal educational and social movement. Lady Mary Wortley Montagu's *Letters* from Turkey, for example, “were influential both as models of epistolary style and as anthropological works” (Somervell, 2011). Our results corroborate these findings in linguistic terms: from the 1760s onward, women initiate changes across linguistic levels diverging from past language use of both women and men—changes which are subsequently adopted by men.

While we have focused more on the informal setting in this paper, it would be interesting to also more deeply investigate how gender-specific change in language use has propagated within more formal registers, such as court proceedings (cf. Degaetano-Ortlieb, 2018) or scientific writing (cf. Degaetano-Ortlieb and Teich, 2019), given enough gender-annotated data. Furthermore, considering that conventionalization seems essential in language change as a precondition for innovation (Bybee, 2010; Schmid, 2015; De Smet, 2016; Teich et al., 2021), it would be worth studying the interplay between convention and innovation from a gender perspective. In addition, given enough data, a network analysis would be intriguing to trace the propagation of innovation across individuals (see, e.g., Sairio, 2009).

Our study offers two main methodological contributions to the analysis of long-term diachronic data, adapting computational modeling to form novel ways of inspecting long-term temporal dynamics of language use. For detection of change, we use diachronic periodization based on Kullback-Leibler divergence, allowing us to determine when changes occur (rather than using pre-defined periods for comparison), using a wide range of features (avoiding pre-selection bias) across linguistic levels, and to derive relevant features of variation in language use from the data at hand. The application of event cascades based on the Multivariate Hawkes Process (usually employed in sociolinguistics to model turn-taking interactions) on long-term diachronic data allows us to model influencer groups of women and men over time. Our results conform with a long-term assumption of women being involved in leading registerial change over time in more informal settings.

A major challenge that has to be faced in diachronic analysis is the representativeness of corpus data. While diachronic corpora are extremely valuable resources and the compilers of such corpora have undertaken immense effort to create these resources in the best way possible, the fact remains that representativeness cannot be achieved as fully as for contemporary synchronic studies (cf. Gries and Hilpert, 2008). Diachronic data is ultimately a finite sample constrained by past data availability. Nevertheless, our findings from the perspective of sociolinguistics corroborate findings across other disciplines, such as literary studies and history, showing how computational sociolinguistic work adds to the scientific endeavor of better understanding the temporal dynamics of change. The methodology of using Kullback-Leibler Divergence to detect change has already been applied to other diachronic corpora and has shown its validity on smaller ones as well.^13^ Regardless of size, spelling variation is also an issue for diachronic corpora. Better methods of spelling normalization are currently being developed (e.g., Hämäläinen et al., 2019), an endeavor that should be pursued further. Future methodological development in dealing with these small but complex datasets should perhaps especially focus on issues of potential bias and outliers, so that they could be alleviated, and so that human analysts would be alerted to particularly sparse or skewed data in specific time periods. In diachronic research, computational analysis should always be complemented with qualitative human analysis, contextualization, and interpretation, as we have striven to do here.

## Data Availability Statement

The *Old Bailey Corpus* v.2.0 is available at: https://hdl.handle.net/11858/00-246C-0000-0023-8CFB-2 (persistent handle). Owing to copyright reasons, access to the *Tagged Corpus of Early English Correspondence Extension* as a whole is restricted to on-site use at the University of Helsinki; however, parts can be made available for research purposes upon request. Data and scripts are made available at: https://github.com/degaetano-ortlieb/frontiersCompSocioLingRegisterGender.

## Author Contributions

SD-O and TS contributed the conception and design of the study. SD-O wrote the first draft of the manuscript, provided the background on register studies, introduced the data-driven periodization by KLD, conducted the KLD analysis, and interrelated the KLD, cascade, and micro-analytical results. TS provided the historical sociolinguistic background and interpretation, information on the TCEECE, data cleaning for the suffixes with SD-O, and micro-analytic inspection of the study, partly with SD-O at the lexical and grammatical levels. YB ran the preliminary Hawkes Process investigations on the TCEECE dataset, adapted the event cascade framework to the long-term gender influence scenario, and conducted the event cascade analyses. All authors contributed to manuscript revision, read, and approved the submitted version.

## Conflict of Interest

The authors declare that the research was conducted in the absence of any commercial or financial relationships that could be construed as a potential conflict of interest.

## Footnotes

^1^also comparing a corpus of scientific texts [RSC (Kermes et al., 2016; Fischer et al., 2020)] to a general English corpus [CLMET (De Smet, 2006; Diller et al., 2010)] to discern change specific to scientific writing.

^2^ https://alraqmiyyat.github.io/OpenITI/

^3^Landing page: OBC (V2.0) https://www.clarin.eu/showcase/old-bailey-corpus-20-1720-1913.

^4^Note that pointwise KLD can also result in negative values, i.e., features have a negative contribution for *A*.

^5^*-able, -age, -al, -ance, -ant, -ary, -ate, -eer, -ence, -ent, -er, -ful, -hood, -ian, -ible, -ic, -ion, -ism, -ist, -ity, -ive, -ize, -less, -ment, -ness, -ology, -ous, -ship, -tude, -ure*.

^6^It is important to stress here that the concept of influence is to be held as likely, and not absolutely ascertained: we are de facto observing correlation, not causation. For example, it may be that all parties are being influenced by the same hidden source with a delay, i.e., early adopters would not be influencing late adopters. When we say that *X* influences *Y*, we mean that their behavior is consistent with that of an event cascade initiated by *X* and continued by *Y*.

^7^In this case we will not directly consider one-to-many influences.

^8^Approximately two thirds of the distinct word forms or types (typically the least frequent forms) cannot be automatically mapped to the *Oxford English Dictionary*, even if we exclude words tagged as foreign or proper nouns (Säily and Mäkelä, 2019). While we do not have similar information for the OBC, it is to be expected that the proportion would be lower there.

^9^That is, 20 years before 1760 diverge from 20 years after 1760 by 1.0 bits, 1/3 more than 20 years before and after 1740.

^10^Past female language use differs from future language use of both men and women, cf. rising tendency of futureF vs. pastF in Figure 5A; future female language use converges to male.

^11^Over the most frequently used words in each period—thus including a good deal of stylistically informative function words. We also tried this with more low-frequency content words, obtaining essentially similar results in terms of overall influence.

^12^Examples are given in their original spelling. Boldface has been added, while italics (if any) are as printed in the letter edition and probably reflect underlining in the original manuscript. PIERCE_028 is the unique identifier of the letter in the corpus.

^13^For example, KLD has been applied to the *Royal Society Corpus* (RSC; Kermes et al., 2016; Fischer et al., 2020) with ~32 million tokens, the *Corpus of Late Modern English Texts* (CLMET; Diller et al., 2010) with ~40 million tokens (see Degaetano-Ortlieb and Teich, 2019) as well as to a small corpus of history texts (CHET; Moskowich et al., 2019) with 40 texts amounting to ~500,000 tokens (see Degaetano-Ortlieb et al., 2019c).
